# First case of coloboma, lens neovascularization, traumatic cataract, and retinal detachment in a young Asian female

**DOI:** 10.1002/ccr3.4743

**Published:** 2021-08-30

**Authors:** Bharat Gurnani, Kirandeep Kaur, Subhashini Sekaran

**Affiliations:** ^1^ Cataract, Cornea and Refractive Services Aravind Eye Hospital and Post Graduate Institute of Ophthalmology Pondicherry India; ^2^ Paediatric Ophthalmology and Strabismus Services Aravind Eye Hospital and Post Graduate Institute of Ophthalmology Pondicherry India; ^3^ DNB Resident Aravind Eye Hospital and Post Graduate Institute of Ophthalmology Pondicherry India

**Keywords:** cataract, coloboma, lens neovascularization, retinal detachment

## Abstract

Lens neovascularization is a very rare entity to be encountered in the clinical practice. It is possibly a result of chronic ocular inflammation due to injury and subsequent uveitis. Early diagnosis and meticulous management can salvage visual acuity in such cases.

## Graphical abstract

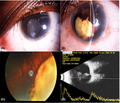

## 1 CASE DESCRIPTION

A young girl post‐stick injury presented with traumatic cataract and lens neovascularization in OS and iris coloboma, keyhole pupil, and cataractous changes in OD. Fundoscopy revealed chorioretinal coloboma OD and retinal detachment on B scan OS. In view of guarded visual prognosis, the patient was advised observation in OU.

**FIGURE 1:**
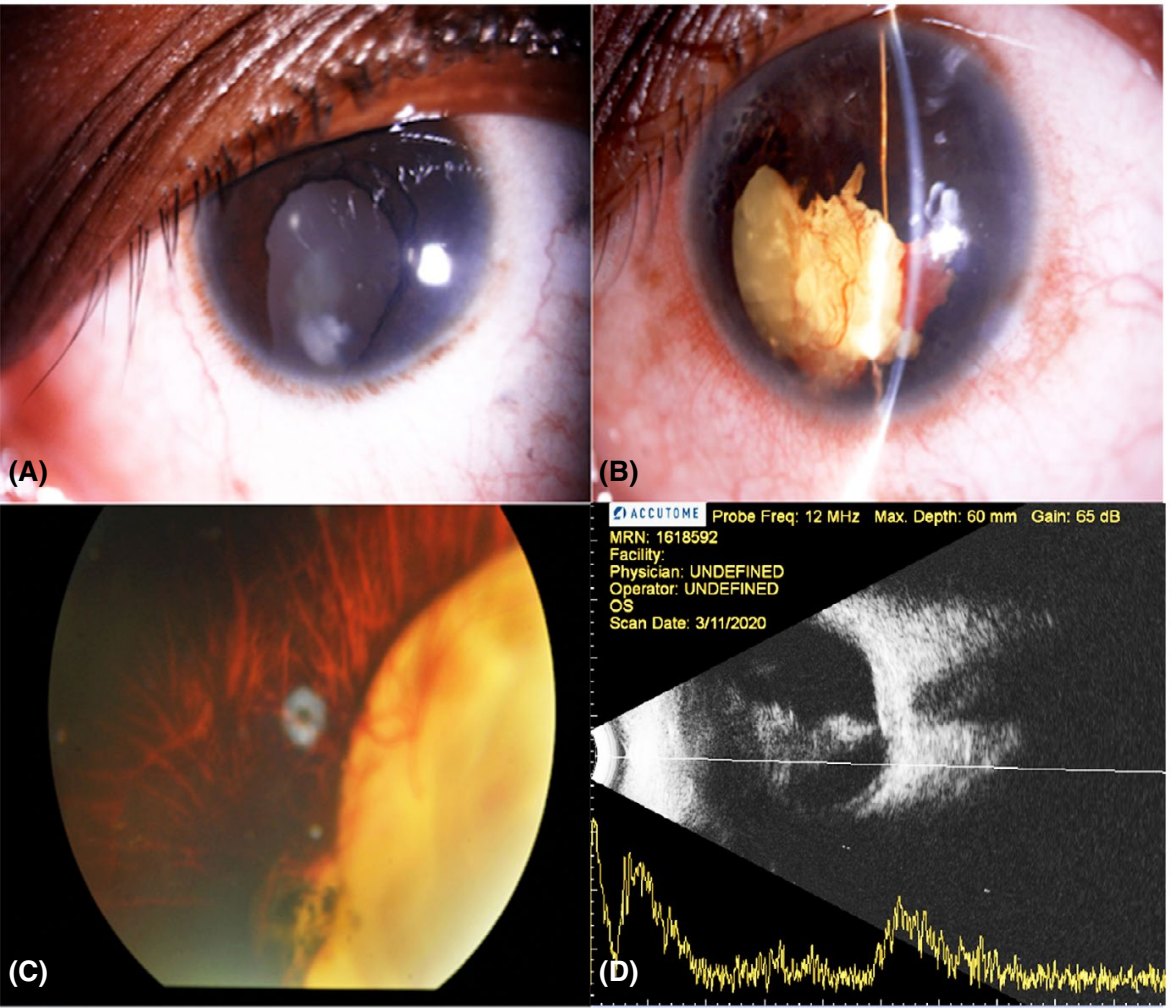
A, Image of the right eye depicting inferior iris coloboma, key whole pupil, and focal cataractous changes. B, Image of the left eye depicting inferior iris coloboma with multiple sphinter tears and patches of iris atrophy, keyhole pupil, and a vascularized membrane in the anterior chamber. C, Fundus image of the right eye depicting chorioretinal coloboma involving the disk and macula. D, B scan image of the left eye depicting lens echoes and moderate to high reflective membranous echoes attached to optic nerve head posteriorly and posterior aspect of lens anteriorly

A 25‐year‐old girl presented with recent onset deterioration of vision in the left eye (OS) in our OPD. She gave the history of trauma with the stick in OS 2 months back. Best corrected visual acuity (BCVA) was hand movements in the right eye (OD) and no perception of light (No PL) in OS. Intraocular pressure was 14 mmHg OD and 4 mmHg OS. Detailed anterior segment examination in OD revealed horizontal jerky nystagmus, inferior iris coloboma, keyhole pupil, and focal cataractous changes (Figure 1A). Ocular examination of OS revealed horizontal jerky nystagmus, circumciliary congestion, shallow anterior chamber, multiple sphinter tears with atrophic iris, irregular pupil, and cataractous lens with numerous discrete blood vessels over the lens capsule (Figure 1B). Fundoscopy in OD revealed chorioretinal coloboma involving the disk and macula (Figure 1C) and was obscured in OS secondary to traumatic cataract. Ultrasound B scan depicted lens echoes and moderate to high reflective membranous echoes attached to optic nerve head posteriorly and posterior aspect of lens anteriorly. The membranous echoes in the posterior segment showed limited mobility (Figure 1D). A diagnosis of iris coloboma, focal cataract, and chorioretinal coloboma was made in OD, and lens neovascularization, traumatic cataract, and retinal detachment were made in OS. This is a very rare and probably the first case of coloboma, cataract, retinal detachment, and lens neovascularization. Surgical intervention was not considered in either eye in view of poor visual prognosis.

Ocular trauma is one of the major yet unrecognized causes of vision loss globally. The complications of blunt ocular trauma such as corneal edema, keratitis, hyphema, secondary glaucoma, cataract, and retinal detachment are well known.^1^ Neovascularization has been reported in various ocular structures such as cornea, iris, anterior chamber angle, retina, and rarely in the lenticular stroma, but lens neovascularization has rarely been reported.^2^

## CONSENT STATEMENT

Published with written consent of the patient.

## CONFLICT OF INTEREST

There are no conflicts of interest.

## AUTHOR CONTRIBUTIONS

BG has made substantial contributions in the acquisition and interpretation of data, analysis drafting, and revision of the manuscript. KK has made substantial contributions in the analysis and interpretation of the data, and in revising the manuscript. SS helped in the data collection of the patient. All authors read and approved the final version of the manuscript and agree to be accountable for all aspects of the work.

## ETHICAL APPROVAL

At our institute case reports, images, and case series are exempted from IRB approval and the research followed the tenets of the Declaration of Helsinki.

## Data Availability

Not applicable. No data are associated with this report.

## References

[1] Cassen JH. Ocular trauma. Hawaii Med J. 1997;56(10):292‐294. 9385749

[2] Kabat AG. Lenticular neovascularization subsequent to traumatic cataract formation. Optom Vis Sci. 2011;88(9):1127‐1132. doi:10.1097/OPX.0b013e31822311e2 21642887

